# Wide-Viewing-Angle Integral Imaging System with Full-Effective-Pixels Elemental Image Array

**DOI:** 10.3390/mi14010225

**Published:** 2023-01-15

**Authors:** Zesheng Liu, Dahai Li, Huan Deng

**Affiliations:** College of Electronics and Information Engineering, Sichuan University, Chengdu 610065, China

**Keywords:** integral imaging, viewing angle, elemental image array, effective pixels

## Abstract

There exists a defect of the narrow viewing angle in the conventional integral imaging system. One reason for this is that only partial pixels of each elemental image contribute to the viewing angle and the others cause image flips. In this paper, a wide-viewing-angle integral imaging system with a full-effective-pixels elemental image array (FEP-EIA) was proposed. The correspondence between viewpoints and pixel coordinates within the elemental image array was built up, and effective pixel blocks and pixels leading to flipping images were deduced. Then, a pixel replacement method was proposed to generate the FEP-EIAs, which adapt to different viewing distances. As a result, the viewing angle of the proposed integral imaging system was effectively extended through the replacement of the pixels, which caused the image flips. Experiment results demonstrated that wide viewing angles are available for the proposed integral imaging system regardless of the viewing distances.

## 1. Introduction

Integral imaging (InIm) is a popular three-dimensional (3D) light-field display technique. Besides its merits of full parallax, quasi-continuous viewpoints, and no so-called vergence-accommodation conflict, the features of small form factor and real 3D depth reconstruction broaden its applications in entertainment, commercial exhibition, medicine, and industrial design [1,2,3].

InIm technology has been developed for over one hundred years; early research was focused on resolving its depth reverse problem [4], while in the last few decades, researchers have been devoted to improving its 3D display performances including the 3D resolution, viewing angle, and depth of field [5,6]. Among them, the extension of viewing angles plays a key role in improving viewing freedom and promoting the commercial use of this technology. Many innovative methods and systems have been reported. For example, InIm systems based on curved lens array [7,8,9] were built to make the parallel reconstructed rays converge to the center and thus increase the public viewing zones of the lens units. Methods using multi-plate lenses [10,11,12] have also been reported; by designing and optimizing the compound lens, the individual field of view (FOV) of each lens unit was extended. Additionally, a high refractive index medium was employed between the elemental image plane and the lens array to increase the deflections of the exit rays [13]. There is another group of methods that reform the system structures in time division or space division multiplexing ways. For instance, in the method proposed by Lee et al. [14], the lens switch is implemented by opening and shutting each lens, so the elemental image covered by each lens was equivalently enlarged and multiple views were spliced to realize the extension of the viewing angle. Kim et al. developed a time-multiplexed sequential projection structure to display two-directional elemental image sets on the same image screen so that the viewing angle was doubled [15]. Sang et al. utilized three groups of directional backlights and a fast-switching LCD panel to multiplex the limited pixels in time division and reached a 120-degree wide viewing angle [16]. In addition, the combination of position-tracking technologies and 3D display technologies provides an important way to overcome the challenges of viewing angle enhancement in the InIm system. A head-tracking InIm system was first presented by Park et al. [17]; the lateral and longitudinal positions of the viewer were captured by the infrared sensors, and suitable perspective images were provided to the viewer at different positions of a large viewing zone to improve the viewing angle effectively. Then, Shen et al. [18] and Dorado et al. [19] proposed some other methods in combination with the computer image generation algorithm named SPOC, which was presented by Martínez-Cuenca et al. [20], to greatly enhance the viewing angle of the InIm system. Xiong et al. [21] proposed a viewing angle enhanced InIm system with partially overlapped viewing zones and achieved a large viewing angle of 120 degrees. The above techniques did increase the viewing angle of InIm systems; however, the high requirements on the precision of the lens structures and the refresh rates of display screens, as well as the requirement of auxiliary devices, were the consequences.

In the previous study, we proposed an InIm 3D display [22] by simply enlarging the pitch of elemental images; the views of all lenses were converged to the center to increase the viewing angle. However, this scheme could only extend the viewing angle at a fixed viewing distance. Additionally, the non-correspondence of the elemental image pitch in both the recording and displaying stages resulted in depth distortion, compared with the original 3D scene. A method with extensions of the effective zones within the EIA [23] was reported which achieved an equivalent wide viewing angle as the convergent scheme, yet the depth distortion caused by the variation in focal length still has to be considered. Moreover, the relations between the pixel alignments of elemental images and the viewpoints distributions were analyzed [24,25], and the viewpoints distribution could be adjusted by controlling the arrangement of elemental images.

Here, a wide-viewing-angle InIm 3D display system adapting to different viewing distances, as well as a method of generating full-effective-pixels EIA (FEP-EIA), are presented. This system has the same small form factor as the conventional InIm 3D display, with the full-effective utilization of the pixels within the EIA. The viewing angle is extended through pixel replacement rather than elemental image zooming so that the advantage of real 3D depth reconstruction remains. Experimental results demonstrate that a much wider viewing angle, compared with the conventional InIm system, is always achievable even if the viewing distance varies.

## 2. Analysis of Viewing Zones and Effective Pixel Block

**Figure 1:**
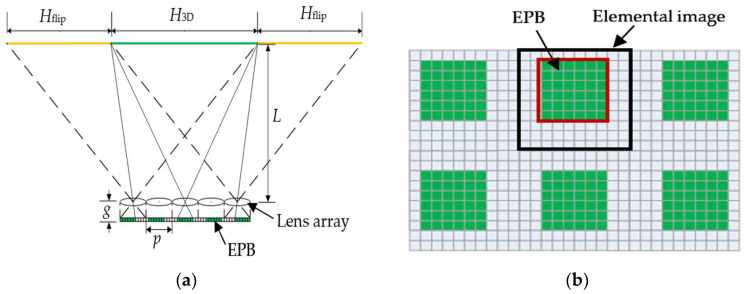
(**a**) Distributions of viewing zone and image flipping zones; (**b**) Effective pixel blocks within the EIA.

In the conventional InIm system, rays emitted from part of the pixels within the EIA are refracted by the lens array laid in parallel with the two-dimensional (2D) display screen to form the viewing zone, as represented by the green line in Figure 1a. Additionally, that emitted from the rest of the pixels are related to the image flipping zones, as denoted by the yellow lines. The width *H*_3D_ and height *V*_3D_ of the viewing zone, as well as the width *H*_flip_ and height *V*_flip_ of the image flipping zones besides the viewing zone, can be, respectively, written as

(1) $$\left\{\begin{array}{l} H_{3D}=\frac{Lp}{g}-(M-1)p\\ V_{3D}=\frac{Lp}{g}-(N-1)p \end{array}\right.$$

and

(2) $$\left\{\begin{array}{l} H_{flip}=(M-1)p\\ V_{flip}=(N-1)p \end{array}\right.,$$

where *p* stands for the pitch of both the lens array and the EIA, *g* is the gap between the lens array and the 2D display screen, and *L* is the viewing distance, which is usually a finite value for the certain size of the display system, between the lens array and the viewer, and *M* and *N* represents the number of lenses in horizontal and vertical direction respectively.

For every single elemental image, there exists an image area that corresponds to the viewing zone, named here as effective pixel block (EPB), as shown in Figure 1b, and the rest of the pixels are associated with the image flipping zones. Assuming that the (*x*, *y*) pixel within the EIA is the origin of the EPB within the (*k*, *l*) elemental image, then the four corners of this EPB can be expressed as the (*x*, *y*), (*x* + Δ*x*, *y*), (*x*, *y* + Δ*y*) and (*x* + Δ*x*, *y* + Δ*y*) pixel, respectively, and the values of *x*, *y*, Δ*x*, and Δ*y* can be calculated through

(3) $$\left\{\begin{array}{l} x=round\left[\frac{(k-1)(L+g)p}{rL}\right]\\ y=round\left[\frac{(l-1)(L+g)p}{rL}\right]\\ \Delta x=round(\frac{gH_{3D}}{L})\\ \Delta y=round(\frac{gV_{3D}}{L}) \end{array}\right.,$$

where *r* denotes the resolution of the elemental images. Therefore, for a specified InIm system with certain parameters of *r, g*, *p*, and *L*, the positions of every EPB can be deduced. By the reutilization of the rest pixels related to the image flipping zones, the total amount of effective information displayed on the system can be increased, which provides a way to extend the viewing zone of the InIm system.

**Figure 2:**
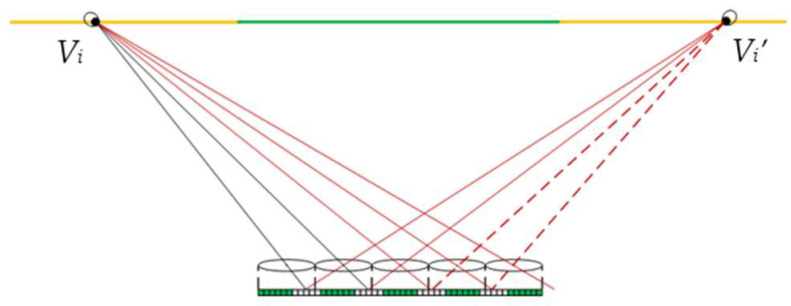
The emergence of the flipping image.

Both the viewing zone and image flipping zones can be considered as the alignment of a set of dense viewpoints, and viewers standing at any viewpoint within the viewing zone can produce a perception of the correct perspective by watching a single pixel from each EPB through its corresponding lens. In case the viewer stands at the viewpoint *V_i_* within the left image flipping zone, his eyes will see, through the corresponding lens, one pixel, respectively, from each of the left two elemental images, as presented by the black lines in Figure 2, while another two pixels can also be seen from the subsequent two elemental images, respectively, through its adjacent lens, as represented by the red lines. In fact, these two pixels belong to the elemental images captured from viewpoint *V_i_^’^* on the right side in the recording stage, as shown represented by the dashed red lines. Therefore, the viewer observes a flipping image with inconsistent parallaxes. In the same vein, the viewer will also observe a flipping image from the right viewpoint *V_i_^’^* which appears in pair with viewpoint *V_i_*.

## 3. Proposed Wide-Viewing-Angle InIm System with FEP-EIA

### 3.1. Structure and Principles

As explained above, only the pixels beyond the range of the EPBs are related to image flipping zones, also the viewpoints within both the left and right image flipping zones are generated in pairs. Thus, we propose a wide-viewing-angle InIm system whose viewing zone is extended by half of the image flipping zones on each side. By means of pixel replacement, the flipping images observed from viewpoints within the original image flipping zones can be compensated with newly imported parallax images, thus the image flips can be apparently suppressed and the viewing zone is extended as well.

**Figure 3:**
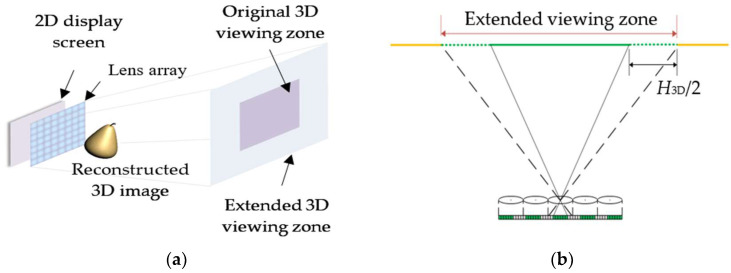
(**a**) Structure and (**b**) extended viewing zone of the proposed InIm system.

The proposed system, consisting of a high-resolution 2D display screen and a lens array, is similar to the conventional InIm system in structure, as shown in Figure 3a. FEP-EIAs for different viewing distances are generated by computer programs and displayed on the 2D display screen; through the modulation of the lens array, viewers can be always offered maximal viewing angles at various viewing distances. Figure 3b shows the extended viewing zone of the proposed system.

### 3.2. Generation of the FEP-EIA

For a specified InIm system, all viewpoints with the same width and height are equidistantly aligned, and the interval Δ*s* between adjacent viewpoints can be expressed as

(4) $$\Delta s=\frac{Lp}{gr}.$$

By assigning the viewpoint located at the top-left corner of the extended viewing zone as the initial one, denoted by *view* (1, 1), the horizontal distance *d_ij-_*_H_ and vertical distance *d_ij-_*_V_ between *view* (*i*, *j*) and *view* (1, 1) can be written as

(5) $$\left\{\begin{array}{l} d_{ij-H}=(j-1)\Delta s\\ d_{ij-V}=(i-1)\Delta s \end{array}\right..$$

**Figure 4:**
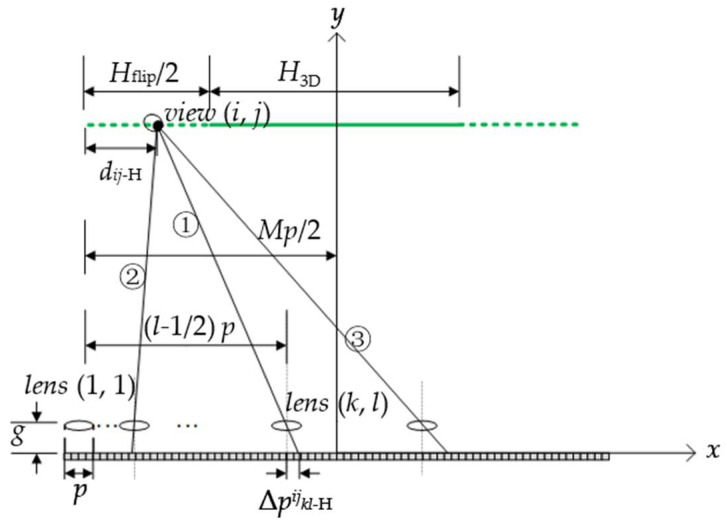
Correspondence between *view* (*i*, *j*) and the pixels displayed on the screen.

The positions of the pixels that *view* (*i*, *j*) captures from the 2D display screen can be determined through the reverse tracing of the rays starting from *view* (*i*, *j*) and passing through the center of each lens, as shown in Figure 4. According to the geometrical relations, the horizontal deviation, denoted by Δ*p^ij^_kl-_*_H_, between the lens center and the pixel hit by the ray through the center of *lens* (*k, l*) can be given by equation

(6) $$\Delta p_{kl-H}^{ij}==\frac{g}{L}\left\{\left(\frac{H_{flip}}{2}+\frac{H_{3D}}{2}-d_{ij-H}\right)-\left[\frac{Mp}{2}-(l-\frac{p}{2})\right]\right\}.$$

Similarly, the vertical deviation Δ*p^ij^_kl-_*_V_ can be given by equation

(7) $$\Delta p_{kl-V}^{ij}==\frac{g}{L}\left\{\left(\frac{V_{flip}}{2}+\frac{V_{3D}}{2}-d_{ij-V}\right)-\left[\frac{Np}{2}-(k-\frac{p}{2})\right]\right\}.$$

Equations (6) and (7) apply to cases where *lens* (*k, l*) is located on the right (case ①) and left (case ②) side of *view* (*i*, *j*), as well as that *lens* (*k, l*) lies to the left (case ①) and right (case ③) side of the central symmetrical axis of the system. According to the calculated values of the horizontal and vertical deviations, the traced pixel shifts to the left for Δ*p^ij^_kl-_*_H_ < 0 and to the right for Δ*p^ij^_kl-_*_H_ > 0. Similarly, it moves up when Δ*p^ij^_kl-_*_V_ > 0 and down when Δ*p^ij^_kl-_*_V_ < 0.

**Figure 5:**
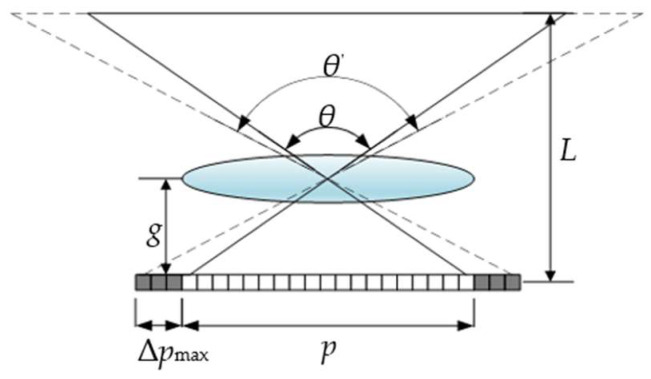
FOV extension for the lens.

In the condition of both |Δ*p^ij^_kl-_*_H_| ≤ *p*/2 and |Δ*p^ij^_kl-_*_V_| ≤ *p*/2, the traced pixels exist within the elemental image covered by *lens* (*k, l*) and can provide the correct perspective for *view* (*i*, *j*), while for either |Δ*p^ij^_kl-_*_H_| > *p*/2 or |Δ*p^ij^_kl-_*_V_| > *p*/2, the traced pixels lie beyond the range of that elemental image and therefore lead to image flips. To avoid this crosstalk and provide *view* (*i*, *j*) with a sequential perspective, a lens array with a larger FOV for each lens is adopted to capture extra perspective information in the recording stage of InIm, as shown in Figure 5. Here, we define Δ*p*_max_ as the value of the maximum element within the two four-dimensional arrays constituted by Δ*p^ij^_kl-_*_H_ and Δ*p^ij^_kl-_*_V_ to ensure that every lens captures a large enough perspective and the FOV set in the recording stage, denoted as *θ*^’^, should be no less than

(8) $$\theta^{\prime }=\arctan\left[\frac{2\Delta p_{\max}+p}{2g}\right].$$

The extended EIA generated with FOV *θ*^’^ for each lens can provide supplementary pixel information for the generation of the FEP-EIA.

**Figure 6:**
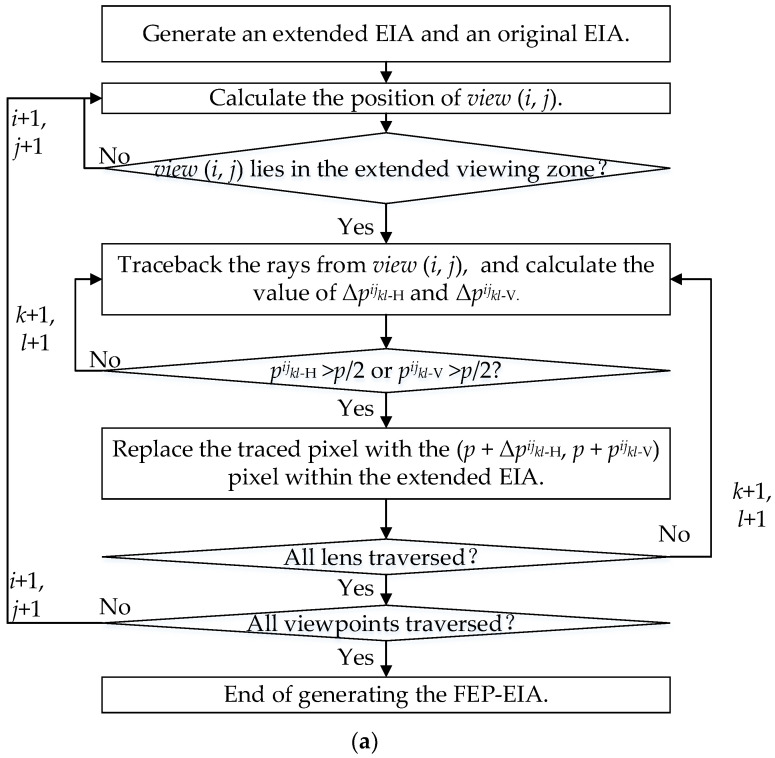

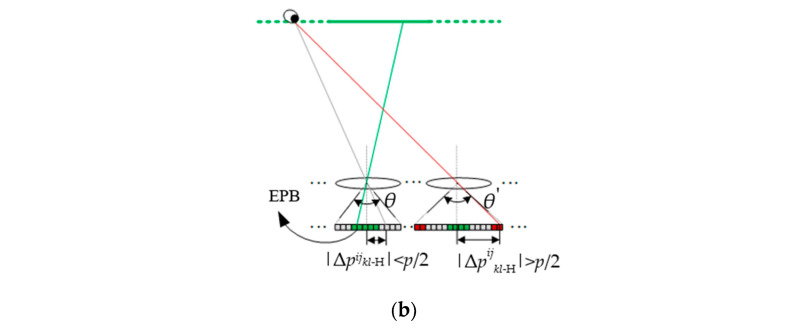
Generation of the FEP-EIA. (**a**) Flowchart of the algorithm; (**b**) Relationships among *view* (*i*, *j*), *lens* (*k*, *l*) and Δ*p^ij^_kl-_*_H_.

Figure 6a shows the flowchart of the algorithm being used to generate the FEP- EIA. At first, an extended EIA is generated with FOV *θ*^’^ for each lens, and then an original EIA with conventional FOV is extracted from the extended EIA. When *view* (*i*, *j*) is located in the original viewing zone (Solid green line), it corresponds to the EPBs within the original EIA, as shown by the green ray in Figure 6b, so these pixels should remain. For the case that *view* (*i*, *j*) lies in the extended viewing zone (Dotted green line), the values of Δ*p^ij^_kl-_*_H_ and Δ*p^ij^_kl-_*_V_ are calculated. If |Δ*p^ij^_kl-_*_H_| ≤ *p*/2 and |Δ*p^ij^_kl-_*_V_| ≤ *p*/2, as denoted by the gray ray, the traced pixels should be kept as well according to the discussion above. When |Δ*p^ij^_kl-_*_H_| > *p*/2 or |Δ*p^ij^_kl-_*_V_| > *p*/2, as denoted by the red ray, the color of the traced pixel is replaced by that of the (*p* + Δ*p^ij^_kl-_*_H_, *p* + Δ*p^ij^_kl-_*_V_) pixel extracted from the (*k*, *l*) elemental image of the extended EIA. After traversing all the viewpoints and lenses, an FEP-EIA is finally generated.

## 4. Experiments and Results

**Figure 7:**
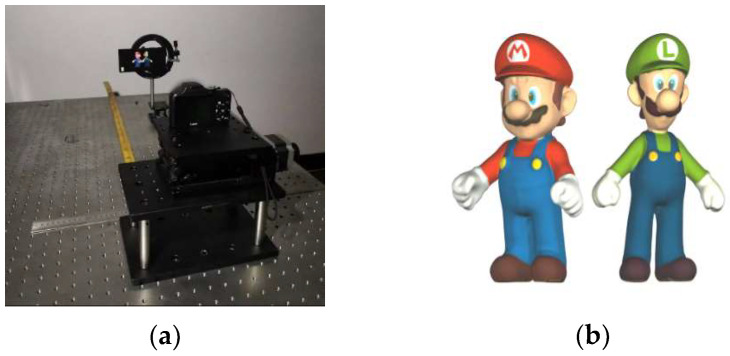
Configurations of the experiment. (**a**) 3D display prototype; (**b**) 3D scene created in software 3ds Max.

A 3D display prototype, as shown in Figure 7a, was constructed to demonstrate the viewing angle extensions of the proposed method. The 2D display screen used here is a smartphone, the SONY XPERIA Z5 PREMIUM, which owns a 5.5 inches TFT-LCD display with a high resolution of 3840 × 2160 pixels and a minuscule pixel pitch of 31.5 µm. To precisely match such a small pixel pitch, a pinhole array rather than the lens array is used as the optical modulator. The detailed parameters are listed in Table 1.

A 3D scene with two popular cartoon characters of “Mario” and “Luigi” was created in the software 3ds Max. Within it, “Mario” stood about 60 mm ahead of “Luigi”, as shown in Figure 7b.

**Figure 8:**
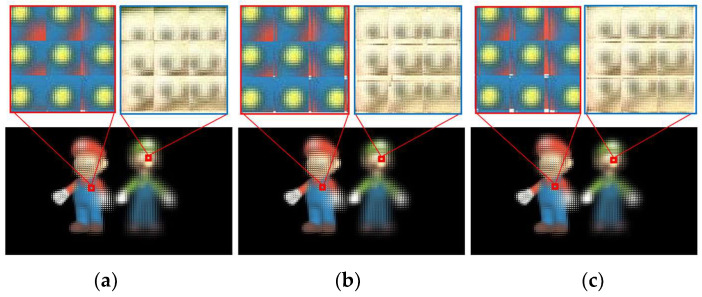
Comparisons of (**a**) Conventional EIA, (**b**) FEP-EIA with *L* = 450 mm, and (**c**) FEP-EIA with *L* = 250 mm.

A conventional EIA, consisting of 128 × 72 elemental images with a resolution of 30 × 30 pixels per image was generated by the conventional InIm method at first, as shown in Figure 8a. Then, two FEP-EIAs were generated with the proposed method, one for the viewing distance of 450 mm, and the other for 250 mm, as shown in Figure 8b,c. From the partially enlarged details, tiny offsets were shown in the elemental images within the three EIAs regarding the same part of the 3D scene. These offsets are actually produced by the pixel replacement of the proposed method, and, in addition, the offsets of the elemental images vary with the viewing distances.

The theoretical viewing angle of the conventional InIm system can be calculated through [22]

(9) $$\theta_{c}=2\arctan\left[\frac{p}{2g}-\frac{p(M-1)}{2L}\right].$$

As shown in Table 2, the theoretical viewing angle of the conventional InIm was 9.3 degrees for the distance of 450 mm, and −2.9 degrees for the distance of 250 mm, respectively. The negative angle indicated that the minimum viewing distance of this experimental InIm system was longer than 250 mm, so that no orthoscopic reconstructed 3D images could be observed at that distance. With the proposed method, however, a viewing angle as larger as 24.2 degrees was theoretically achievable for any distance.

**Figure 9:**
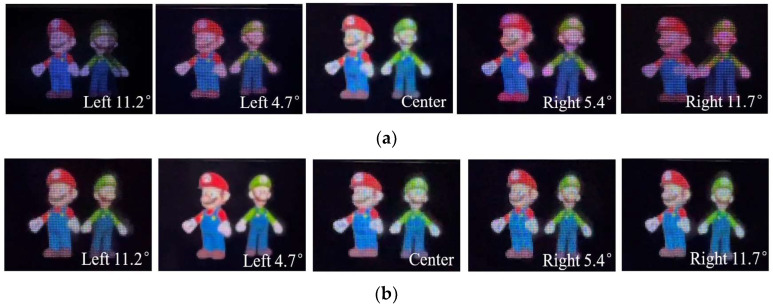
Captured 3D images for (**a**) the conventional EIA and (**b**) the FEP-EIA with *L* = 450 mm.

The EIAs shown in Figure 8a,b were displayed on the 2D screen successively, and at the distance of 450 mm, a camera was used to record the 3D images from different angles. Orthoscopic 3D images, as shown in Figure 9a, were obtained continuously from the left 4.7 degrees to the right 5.4 degrees with the conventional EIA, while for the proposed method, the viewing angle was extended to 22.9 degrees which was basically consistent with the theoretical result, as shown in Figure 9b. Moreover, continuous parallaxes were obtained in the whole range of the viewing angle, and more perspective 3D information was presented, compared with Figure 9a.

**Figure 10:**
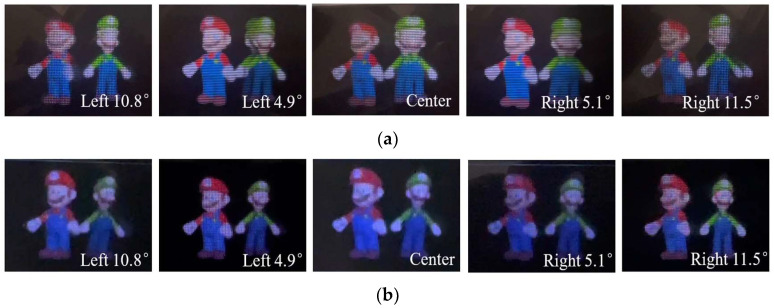
Captured 3D images for (**a**) the conventional EIA and (**b**) the FEP-EIA with L = 250 mm.

A similar experiment was implemented for the viewing distance of 250 mm. It is obvious that with the conventional InIm, orthoscopic 3D images were barely visible from any angles, as shown in Figure 10a. In contrast, the proposed method helped to obtain the integrated 3D images with continuous parallax in the range from the left 10.8 degrees to the right 11.5 degrees, and the reconstructed 3D images were as good as that obtained at the distance of 450 mm. These results demonstrated that the proposed method was capable of extending the 3D viewing angle regardless of the viewing distances, and there were no depth distortions in the reconstructed 3D images. The brightness changes in different perspective images, as shown in Figure 9 and Figure 10, mainly come from the influences of environmental illuminations and the nonuniform distributions of the light intensities in the central and side areas in front of the display screen.

Although the use of the pinhole array decreased the brightness of the reconstructed 3D image to a certain extent, this can be improved with the use of lens array in the future. Additionally, on the basis of the proposed method, the viewing angle can be further enhanced by adjusting the system parameters, for example, using a lens array with larger pitches and putting the lens array closer to the pixel plane. For the system with relatively large viewing angles, the impact of aberrations is no longer negligible, so the pre-correction of aberrations should be imported into the improvement of the proposed method in our following study.

## 5. Conclusions

In summary, a wide-viewing-angle InIm system with a full-effective-pixels elemental image array was presented by replacing the related pixels. The image flips were dramatically suppressed and pixel utilization of the elemental image array was greatly increased, thereby the effective viewing angle was extended. The experimental results demonstrate that significantly enlarged viewing angles were available at different viewing distances. Compared with the conventional viewing angle enhancement methods, the proposed method increases the viewing angle through image processing rather than reformations to the optical structures, thus the compact structure, known as one of the advantageous features of the InIm system, is retained. Moreover, a full-effective-pixels elemental image array can be rapidly generated with just one recording process and a few pixel replacements. In combination with eye tracking or head tracking devices in the future, a wide viewing angle can be readily achievable with the moving of the observer. This will have a positive result, promoting the applications of InIm in mobile and medical terminals.

## Figures and Tables

**Table 1:** Configurations of the experiment.

Components	Parameters	Specifications
2D display screen	Product model	SONY XPERIA Z5 PREMIUM
	Screen size	5.5 inches
	Resolution	3840 × 2160 pixels
	Pixel pitch	31.5 µm
Pinhole array	Pitch of the pinhole	0.946 mm
	Number of pinholes	128 × 72
	The gap between the 2D display and the pinhole array	2.2 mm
EIA generated by the conventional method	Resolution per elemental image	30 × 30
	Number of elemental images	128 × 72

**Table 2:** Comparisons of the viewing angles.

Viewing Distances	Methods	Viewing Angle Calculated in Theory	Viewing Angle Measured with Experiments
450 mm	Conventional InIm	9.3^°^	10.1^°^
	The proposed method	24.2^°^	22.9^°^
250 mm	Conventional InIm	−2.9^°^	N/A
	The proposed method	24.2^°^	22.3^°^

## Data Availability

Data is contained within the article.
